# Differential expression of the aryl hydrocarbon receptor pathway associates with craniofacial polymorphism in sympatric Arctic charr

**DOI:** 10.1186/s13227-015-0022-6

**Published:** 2015-09-16

**Authors:** Ehsan Pashay Ahi, Sophie S. Steinhäuser, Arnar Pálsson, Sigrídur Rut Franzdóttir, Sigurdur S. Snorrason, Valerie H. Maier, Zophonías O. Jónsson

**Affiliations:** Institute of Life and Environmental Sciences, University of Iceland, Sturlugata 7, 101 Reykjavik, Iceland; Biomedical Center, University of Iceland, Vatnsmýrarvegur 16, 101 Reykjavik, Iceland

## Abstract

**Background:**

The developmental basis of craniofacial morphology hinges on interactions of numerous signalling systems. Extensive craniofacial variation in the polymorphic Arctic charr, a member of the salmonid family, from Lake Thingvallavatn (Iceland), offers opportunities to find and study such signalling pathways and their key regulators, thereby shedding light on the developmental pathways, and the genetics of trophic divergence.

**Results:**

To identify genes involved in the craniofacial differences between benthic and limnetic Arctic charr, we used transcriptome data from different morphs, spanning early development, together with data on craniofacial expression patterns and skeletogenesis in model vertebrate species. Out of 20 genes identified, 7 showed lower gene expression in benthic than in limnetic charr morphs. We had previously identified a conserved gene network involved in extracellular matrix (ECM) organization and skeletogenesis, showing higher expression in developing craniofacial elements of benthic than in limnetic Arctic charr morphs. The present study adds a second set of genes constituting an expanded gene network with strong, benthic–limnetic differential expression. To identify putative upstream regulators, we performed knowledge-based motif enrichment analyses on the regulatory sequences of the identified genes which yielded potential binding sites for a set of known transcription factors (TFs). Of the 8 TFs that we examined using qPCR, two (*Ahr2b* and *Ap2*) were found to be differentially expressed between benthic and limnetic charr. Expression analysis of several known AhR targets indicated higher activity of the AhR pathway during craniofacial development in benthic charr morphotypes.

**Conclusion:**

These results suggest a key role of the aryl hydrocarbon receptor (AhR) pathway in the observed craniofacial differences between distinct charr morphotypes.

**Electronic supplementary material:**

The online version of this article (doi:10.1186/s13227-015-0022-6) contains supplementary material, which is available to authorized users.

## Background

**Fig. 1:**
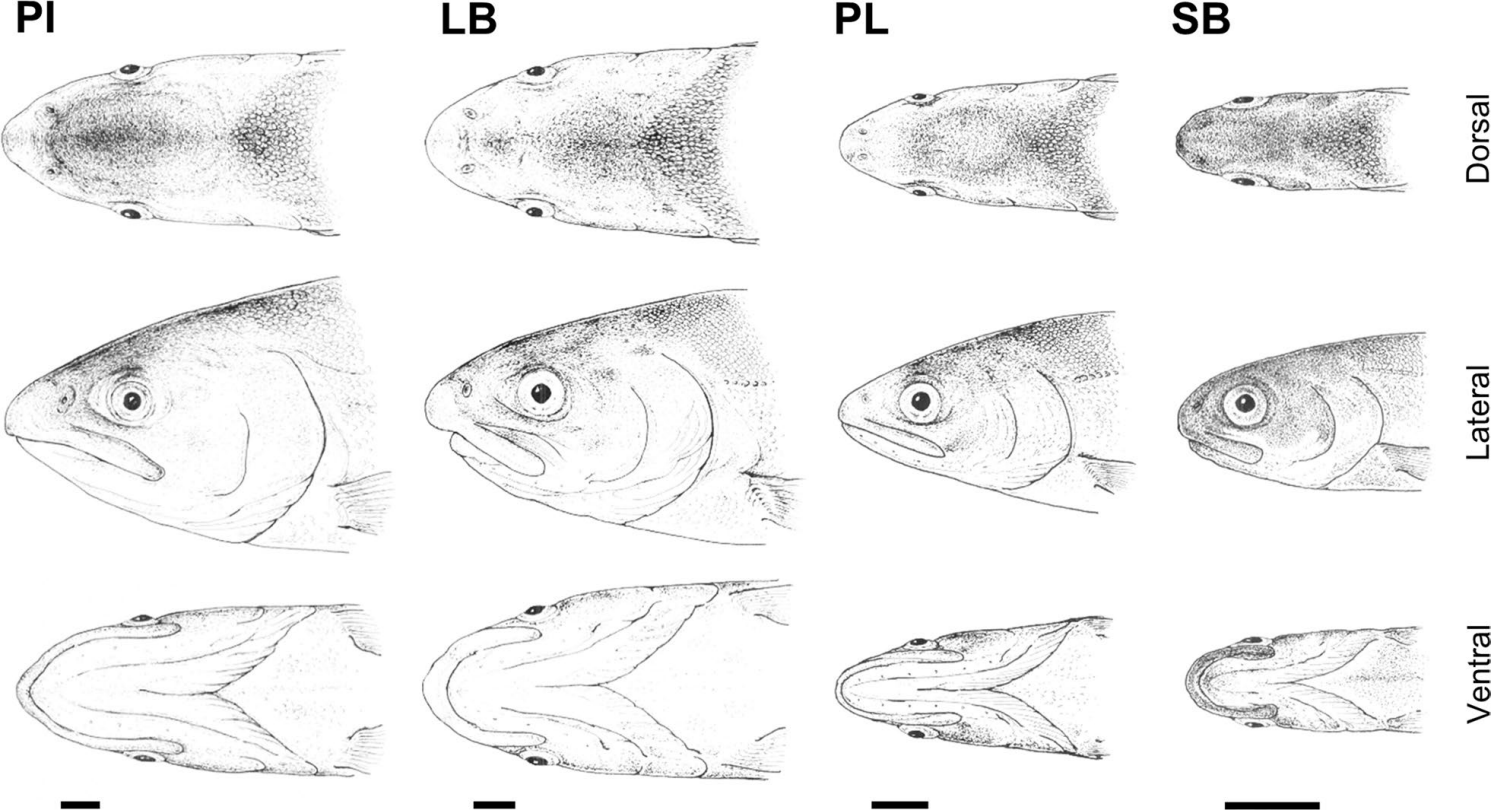
Drawings of adult heads of the four Arctic charr morphs in Thingvallavatn, Iceland. The two limnetic morphs, piscivorous (PI) and planktonivorous (PL) have terminal mouths and pointed snouts and the two benthic morphs, large benthivorous (LB) and small benthivorous (SB) have blunter snouts and more sub-terminal mouths. *Scale bars* represent 1 cm. (drawings by Eggert Pétursson)

Adaptive diversification of craniofacial structures in teleost fish offers a remarkable opportunity to study niche specialization in vertebrates [1]. The formation of craniofacial elements begins at early stages of development through complex and dynamic molecular programs within the tissues bearing relevant patterning information [2]. At the forefront of efforts to understand the underlying developmental and evolutionary mechanisms, species such as zebrafish and cichlids have been extensively studied [3, 4]. Addressing similar questions in non-model species is becoming commonplace due to recent advances in molecular techniques such as large-scale gene expression (transcriptome) profiling and related bioinformatics [5–8]. Among salmonid fishes, the polymorphic Icelandic Arctic charr (*Salvelinus alpinus*) in Lake Thingvallavatn is an outstanding system to investigate intraspecific variation of craniofacial structures [9, 10]. Four distinct subpopulations of Arctic charr are readily distinguishable in this lake based on their genetic, ecological, behavioural and morphological differences [9–15]. Two benthic morphs, large benthivorous (LB) and small benthivorous (SB), have an overshot or subterminal mouth, whereas two limnetic morphs, planktivorous (PL) and piscivorous (PI), have a terminal mouth and a higher number of gill rakers (Fig. 1) [10]. Results obtained in laboratory rearing experiments have suggested a genetic basis as well as a combination of maternal and environmental factors contributing to this divergent trophic morphogenesis [16–18]. We propose that these genetic differences should be detectable as differential transcriptional dynamics of genes involved in craniofacial skeletal development and be reflected later in different, morph-specific phenotypes [19].

In cichlids, transcriptional changes of major signalling pathways during early craniofacial development have been attributed to phenotypic novelties in trophic architecture [20, 21]. Associations between heterochronic shifts in the expression levels of individual skeletogenic genes and distinct craniofacial morphogenesis were indicated in several fish species [22–24]. However, it is not always the case that expression differences of a specific gene result in changes in its protein levels and subsequent phenotypic effects [25]. Instead, looking for transcriptional differences in sets of functionally related genes with conserved co-expression connectivity might provide more reliable evidence concerning the involvement of genes in a specific biological process [26]. More recently, similar efforts have been made to define co-expression gene networks involved in adaptive phenotypic divergence of feeding structures in an East African cichlid species [27] and two salmonid species (Arctic charr and whitefish) [19, 28]. We previously identified a conserved co-expression network consisting of genes involved in extracellular matrix (ECM) organization and skeletogenesis, which was more expressed in the developing craniofacial elements of benthic than in limnetic Arctic charr morphs [19]. Many of these genes are known as downstream targets of several inter-connected regulatory pathways [19]. It is possible that the molecular mechanisms underlying induction of this gene network also affect (activate/repress) other genes and possibly co-expressed networks of genes involved in craniofacial development and skeletogenesis. The identification of groups of genes with tight positive or negative co-expression could provide clues about upstream molecular pathways [29, 30] and even causative genetic changes.

In this study, we investigated transcriptional profiles in developing heads of benthic and limnetic Arctic charr morphs focusing on the period when key elements of the feeding structures are being formed, and, notably, a period when the basis of the benthic–limnetic distinction may be laid [19]. Using transcriptome data of contrasting Arctic charr morphs and through a stepwise approach assisted by co-expression data from mammalian species, we identified genes with higher expression during the morphogenesis of limnetic charr heads. Furthermore, to identify potential upstream regulators, we performed knowledge-based motif enrichment analyses on the regulatory sequences of a network of co-expressed genes (including the identified genes) to predict binding sites for known transcription factors (TFs). Finally, we found *Ahr2b* and other components of a signalling pathway mediated by aryl hydrocarbon receptor (AhR) to be differentially expressed between benthic and limnetic charr, which is consistent with the AhR pathway being a regulator of morph-specific, transcriptional dynamics. These results provide a glimpse into the regulatory networks that seem to shape craniofacial traits in Arctic charr and possibly other teleosts.

## Methods

### Fish stocks and sampling

We sampled sexually ripe adults of the four sympatric morphs from Lake Thingvallavatn; planktivorous (small limnetic) (PL), piscivorous (PI), small benthivorous (SB) and large benthivorous (LB) morphs, and a limnetic-like aquaculture stock (AC) from the Hólar breeding programme [31]. For each morph, eggs from several females were pooled and fertilized using milt from several males. Eggs were reared at approximately 5 °C in hatching trays (EWOS, Norway) under constant water flow and in complete darkness at Holar College experimental facilities in Verið, Sauðárkrókur. The water temperature was recorded twice daily and the average was used to estimate the relative age of the embryos using tau-somite (*τ*_s_) units, defined as the time it takes for one somite pair to form at a given temperature [32]. All sampling from the wild as well as rearing was performed according to Icelandic law and with appropriate permission.

### Genes, databases and overrepresentation analysis

Based on an extensive literature survey and analyses of transcriptome data from early embryonic stages of contrasting Arctic charr morphs (unpublished), we selected our initial sets of candidate genes (see “Results” section) that play a role in craniofacial morphogenesis and/or skeletogenesis, for gene expression profiling. We also checked whether the orthologues of the candidate genes show co-expression relationship in data from mammalian species using COXPRESdb (http://coxpresdb.jp/) version 6.0 [33]). To predict the potential regulators of the genes with reduced expression levels during benthic head development, TF enrichment analysis was conducted using the list of >200 genes co-expressed with *Chd4, Cspp1* and *Dlg1* in humans and mice, as well as the WEB-based GEne SeT AnaLysis Toolkit (WebGestalt) v2 [34]. The involvement of the AhR was investigated by profiling the expression of 12 genes responsive to the pathway which also have been shown to be expressed during craniofacial development in fish and/or involved in vertebrate skeletal formation (Additional file 1: Table S1).

### RNA isolation, cDNA synthesis and primer design for qPCR

We studied embryos of five Arctic charr morphs, collected at 6 tau-somite stages (178, 200, 216, 238, 256 and 275 *τ*_s_). This is the period during which the morphs start developing their distinct craniofacial morphologies [35]. The time points span early craniofacial cartilage formation, 178 (*τ*_s_), to a pre-hatching stage, when ossification of key trophic elements of the jaws has started at 275 (*τ*_s_). The embryos were preserved in RNA-later solution (Ambion) at −20 °C and later dechorionated and decapitated by applying a scalpel vertically in front of the pectoral fin under the light microscope (Leica S6E). For each morph and time-point, two RNA extraction replicates were obtained, each one deriving from six heads. Extractions were conducted in TRI Reagent (Sigma). The heads were homogenized with a disposable Kontes Pellet Pestle Cordless Motor tissue grinder (Kimble Kontes). RNA was extracted according to the manufacturer’s instructions, and DNA contamination was removed by DNase treatment (New England Biolabs). The quantification and quality control of RNA were performed using a NanoDrop ND-1000 UV/Vis-Spectrophotometer (NanoDrop Technologies) and agarose gel electrophoresis. cDNA was synthesized with 1 µg of RNA in a total reaction volume of 20 µl using the High Capacity cDNA RT kit (Applied Biosystems). cDNA was diluted threefold in nuclease-free water for further use in qPCR and several samples without addition of reverse transcriptase were prepared to confirm the absence of genomic DNA. An assembly of the Arctic charr transcriptome [36] was used for qPCR primer design. It is important to note that a whole genome duplication event occurred before the radiation of the salmonid family producing paralogues, most of which are retained in Arctic charr [37]. In this study, we were not focusing on differences between paralogues in expression or function and therefore designed primers to be non-paralogue specific. The only exception is *Ahr2b* where we looked at differential expression of two paralogues as discussed below. To identify the conserved exon/intron boundaries of the candidate genes, the assembled charr contigs were aligned to the genomic sequences of salmon orthologs (retrieved from the salmon database, SalmonDB [38]) using the NCBI Spidey software (http://www.ncbi.nlm.nih.gov/spidey). Primers were designed using Primer Express 3.0 software (Applied Biosystems, Foster City, CA, USA) and checked for self-annealing, hetero-dimers and hairpin structures by OligoAnalyzer 3.1 (Integrated DNA Technology) (Additional file 2: Table S2).

### Real-time quantitative PCR and analysis of expression data

Real-time PCR was performed in 96-well PCR plates on an ABI 7500 real-time PCR System (Applied Biosystems) using Fermentas Maxima SYBR Green qPCR Master Mix (2x) (Fermentas). Each biological replicate was run in duplicate together with no-template control (NTC) in each run for each gene. The PCR was run with a 2 min hold at 50 °C and a 10 min hot start at 95 °C followed by the amplification step for 40 cycles of 15 s denaturation at 95 °C and 1 min annealing/extension at 60 °C. A dissociation step (60–95 °C) was performed at the end of the amplification phase to identify a single, specific melting temperature for each primer set. Primer efficiency values (E) were calculated through the LinRegPCR v11.0 programme (http://LinRegPCR.nl) [39] analysing the fluorescence data from the exponential phase of PCR amplification for each primer pair (Additional file 2: Table S2) and the cut-off was 0.9. The difference between Cq values (ΔCq) of the reference genes and the target genes was calculated for each gene t; ΔCq_target_ = Cq_target_ − Cq_reference_. The geometric mean of Cq values of two validated craniofacial reference genes, *Actb* and *If5a1*, was used for ΔCq calculations [40]. All samples were then normalized to the ΔCq value of a calibrator sample to obtain a ΔΔCq value (ΔCq_target_ − ΔCq_calibrator_). For visual comparisons of expression levels among the developmental time-points and morphs, the biological replicate with the lowest expression (highest ΔCq) was used as a calibrator sample. Relative expression quantities (RQ) were calculated based on the expression level of the calibrator sample (*E*^−ΔΔCq^) [41]. The RQ values were then transformed to logarithmic base 2 values (or fold differences; FD) [42] for statistical analysis. A two-way ANOVA followed by Tukey’s HSD post hoc tests were implemented to examine the effects of morph, time, and morph-by-time interaction on expression of the candidate genes as well as morph-specific expression difference for each gene. To assess similarity in expression patterns of the genes, Pearson correlation coefficients (*r*) were calculated for all gene pairs using the data from 5 morphs at 6 time-points (*df* = 28). To study the relationships between putative regulators and candidate genes, we calculated the correlation (Pearson and Kendall) between all candidate genes (including AhR targets) and the 9 potential regulators (8 predicted TFs and *Foxq1*, an AhR-regulated TF). Next, we tested whether the correlation coefficients varied between TFs or gene categories (higher, lower or equally expressed in benthic morphs), using ANOVAs and non-parametric. R (http://www.r-project.org) was used for all statistical analysis [43].

### Whole-mount in situ hybridization (WISH)

Whole-mount in situ hybridization was performed following a standard procedure for Atlantic salmon [44]. Embryos from a time-point (238 *τ*_s_) representing the early craniofacial bone and cartilage formation were fixed in 4 % (m/v) paraformaldehyde/PBS, dehydrated in a graded methanol series and stored in 100 % methanol. Primers designed for cDNA of selected Arctic charr genes (*Ahr2b*, *Apaf1*, *Cfl1*, *Foxq1*, *Jup, Chd4, Cspp1, and Dlg1*) generated PCR products of around 0.4–0.6 kbp (Additional file 2: Table S2) which were cloned into pCR4-TOPO vector (Invitrogen) and transcribed to antisense/sense digoxigenin (DIG)-labelled cRNA probes with T3 or T7 polymerases (Roche). Five or six embryos from each of the two morphs (PL and SB) were used for in situ hybridization at every relative age. After rehydration and dechlorination, embryos were treated with 20–40 µg/ml proteinase K (New England Biolabs) for 22–60 min, depending on the relative ages. The hybridization was performed with 1 µg/ml (DIG)-labelled RNA probes at 70 °C for 12 h, after which the embryos were incubated with alkaline phosphatase-conjugated anti-digoxigenin antibody (Roche) at 4 °C overnight. The hybridization signals were visualized after 3–12 h incubation at 4 °C using NBT/BCIP (Roche), depending on the expression levels of the genes. The specificity of the antisense probes was also verified by running control experiments with sense probes. Samples were imaged on a Leica MZ10 binocular microscope (Leica).

## Results

### A group of transcriptionally correlated genes shows higher expression in the head of limnetic than benthic morphs

**Fig. 2:**
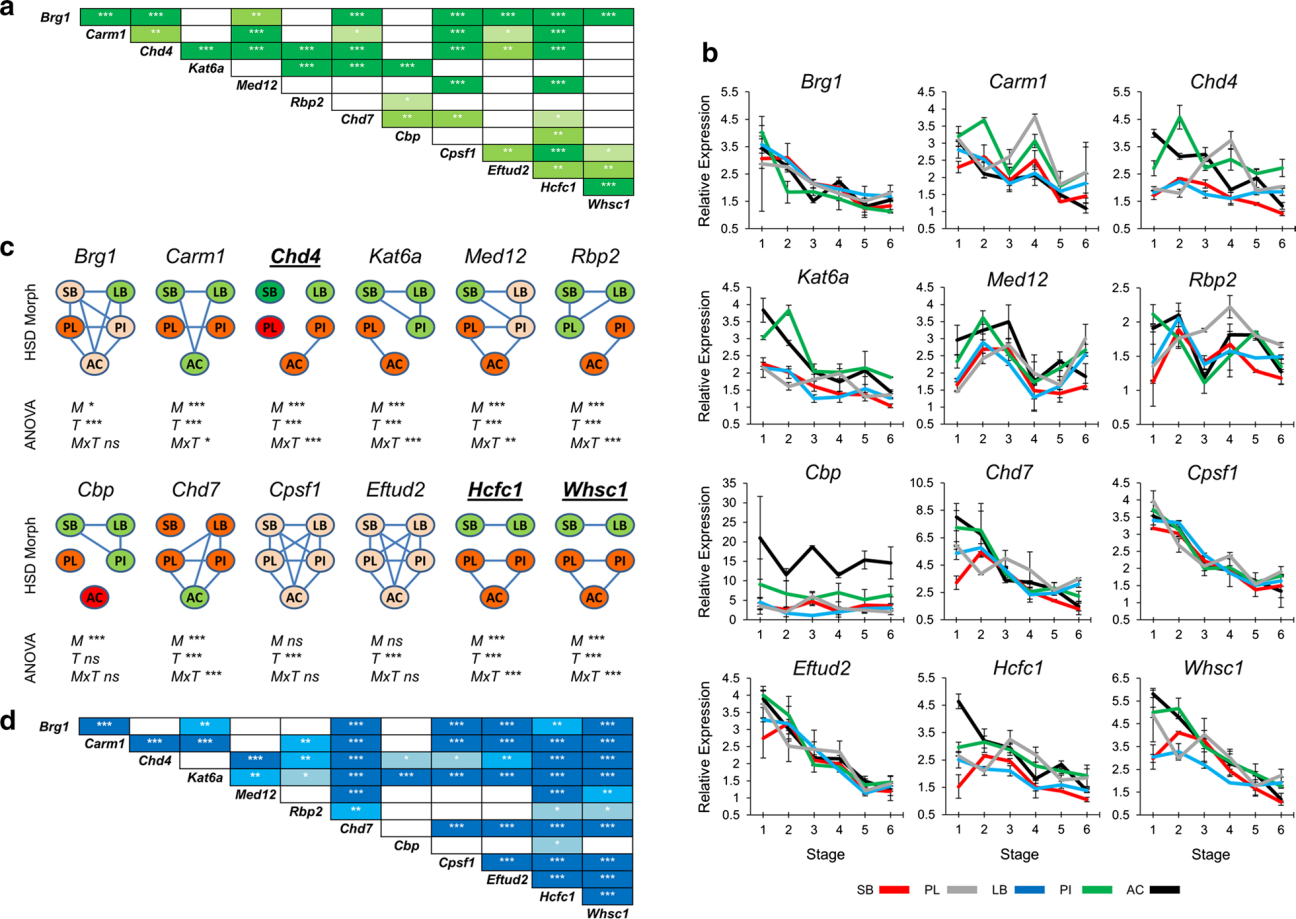
Expression of *Chd4*, *Hcfc1* and *Whsc1* was higher in developing heads of limnetic Arctic charr morphs. **a** Expression correlation of the first twelve candidate genes based on mutual rank (MR) analysis from COXPRESdb in mammalian species. The stronger correlations represented in *darker green shading* and with *one*, *two* and *three asterisks* indicating <2000, <1000 and <500 MR numbers, respectively. **b** Relative expression of the twelve genes in developing heads of SB, LB, PI, PL and AC at six stages. *Error bars* represent standard deviation calculated from two biological replicates where each biological replicate contains a homogenate of six heads. **c** Relative expression ratios were subjected to an analysis of variance (ANOVA) to test for expression differences among five Arctic charr morphs and six stages (*M* morph; *T* stage (time); *MxT* morphs by time effects; *P* values of <0.05, 0.01 and 0.001 are indicated by *one*, *two* and *three asterisks*, respectively). Subsequently, a post hoc Tukey’s HSD test was performed to analyse the expression of candidates among the morphs. *Green* to *red* colour gradient of morphs represent low to high expression levels and morphs with no *connecting line* have significantly different expression (*α* = 0.05). The *bold* and *underlined* genes displayed lower expression levels in the benthic morphs. **d** Pearson correlation coefficient (*r*) was used to assess the pairwise expression similarity between the candidate genes during craniofacial development. *Blue* represents positive expression correlation (the colour gradient showing critical values of *r*, 2-tail; *df* = 28). *P* values of <0.05, 0.01 and 0.001 are indicated by *one*, *two* and *three asterisks*, respectively

We began by studying the expression of six genes (*Brg1, Carm1, Chd4, Kat6a, Med12* and *Rbp2*), which are known to play a role in early craniofacial morphogenesis and/or skeletal differentiation [45–51]. Preliminary mRNA-seq data indicated higher expression of these genes during early embryonic stages in limnetic than in benthic morphs (Gudbrandsson et al. unpublished), and according to COXPRESdb [33] they show co-expression in mammalian species (Fig. 2a). By further examining the co-expression of these genes with other genes from mammals, we found six more genes (*Cbp, Chd7, Cpsf1, Eftud2, Hcfc1* and *Whsc1*), which show conserved co-expression and are all known to play a role in craniofacial skeletal morphogenesis [46, 52–56] (Fig. 2a). To characterize the expression of these 12 candidate genes in the developing head of Arctic charr, we profiled their relative expression in the heads of five morphs (SB, LB, PI, PL and AC) with qPCR, at six consecutive time points, spanning early craniofacial bone and cartilage formation to the prehatching stage (Fig. 2b). The expression of all genes, except *Cbp*, varied significantly over time, showing a general trend of gradual reduction during head development. All genes except *Cpsf1* and *Eftud2* were also expressed at different levels between morphs. However, only three genes, i.e. *Chd4*, *Hcfc1* and *Whsc1*, showed lower expression in both benthic morphs (SB and LB) compared to the limnetic morphs (AC, PI, PL) (Fig. 2c). The benthic–limnetic expression difference was markedly larger for *Chd4* than the two other genes within several developmental time-points (Fig. 2b). A positive correlation of expression levels between most of the candidates, including *Chd4, Hcfc1* and *Whsc1,* was verified in Arctic charr (Fig. 2d).

**Fig. 3:**
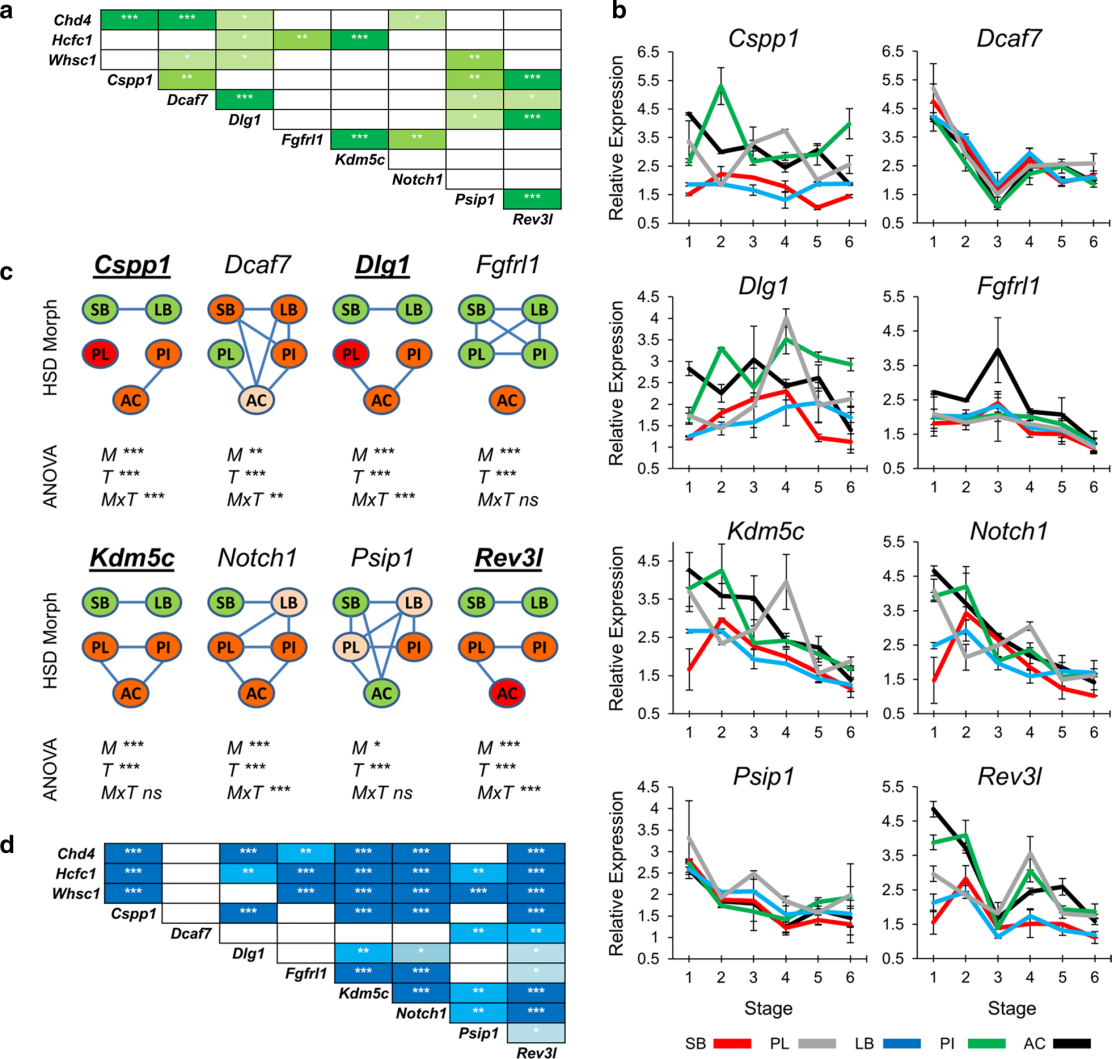
Expression of *Cspp1, Dlg1, Kdm5c* and *Rev3* *l* was higher in developing heads of limnetic than benthic Arctic charr morphs. **a** Expression correlation of the additional eight candidate genes based on mutual rank (MR) analysis from COXPRESdb in mammalian species. The stronger correlations represented in *darker green shading* and with *one*, *two* and *three asterisks* indicating <2000, <1000 and <500 MR numbers, respectively. **b** Relative expression of the genes in developing heads of SB, LB, PI, PL and AC at six stages. *Error bars* represent standard deviation calculated from two biological replicates where each biological replicate contains a homogenate of six heads. **c** Relative expression ratios were subjected to an analysis of variance (ANOVA) to test for expression differences among five Arctic charr morphs and six stages (*M* morph; *T* stage (time); *MxT* morphs by time effects; *P* values of <0.05, 0.01 and 0.001 are indicated by *one*, *two* and *three asterisks*, respectively). Subsequently, a post hoc Tukey’s HSD test was performed to analyse the expression of candidates among the morphs. *Green* to *red* colour gradient of morphs represent low to high expression levels and morphs with no *connecting line* have significantly different expression (*α* = 0.05). The *bold* and *underlined* genes displayed lower expression levels in the benthic morphs. **d** Pearson correlation coefficient (*r*) was used to assess the pairwise expression similarity between the candidate genes during craniofacial development. *Blue* represents positive expression correlation (the colour gradient showing critical values of *r*, 2-tail; *df* = 28). *P* values of <0.05, 0.01 and 0.001 are indicated by *one*, *two* and *three asterisks*, respectively

With the aim of identifying more co-expressed genes with potentially reduced expression in the heads of benthic morphs, we extended the expression analysis by including eight more genes. Seven genes were selected based on co-expression with *Chd4, Hcfc1* and *Whsc1* in mammals (using COXPRESdb [33]) (Fig. 3a) and involvement in vertebrate craniofacial development [57–63]. An eighth candidate, *Rev3l,* was added to the study due to its vital role in early embryonic patterning and craniofacial formation [64] and co-expression with four of the new candidates in mammals (although not *Chd4, Hcfc1* and *Whsc1*) (Fig. 3a). We found that expression of all eight genes differed significantly between morphs and over time, and four of them, i.e. *Cspp1*, *Dlg1*, *Kdm5c* and *Rev3l*, showed lower expression in both benthic morphs compared to all the limnetic morphs (Fig. 3b, c). The benthic–limnetic expression difference was larger for *Cspp1* and *Dlg1* than the two other genes within several developmental time points (Fig. 3b). Again the expression of most of these eight genes was positively correlated with *Chd4*, *Hcfc1* and *Whsc1* from the previous step (Fig. 3d). Taken together, these results show significantly higher expression of 7 genes out of the 20 tested in developing heads of limnetic morphotypes as well as their positive expression correlation during the developmental period under study.

### Potential upstream regulators of the co-expressed genes

**Fig. 4:**
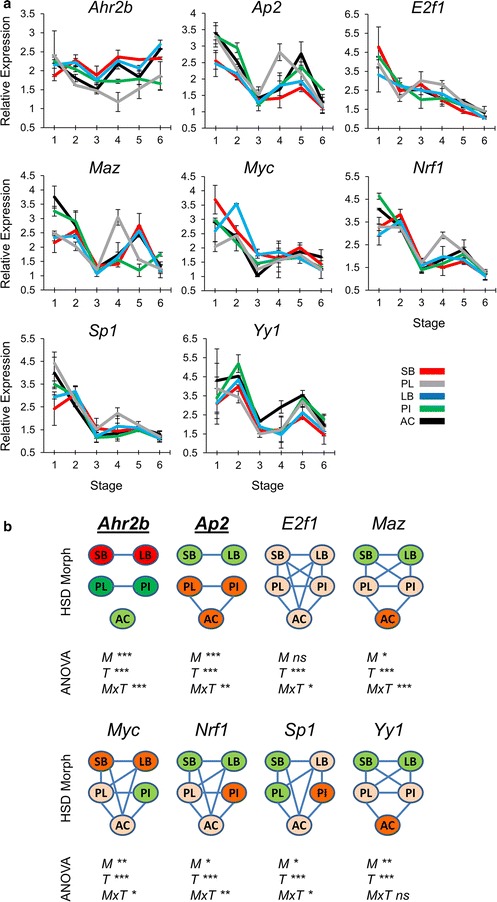
Differential expression of *Ahr2b* and *Ap2* in developing heads of Arctic charr morphs. **a** Relative expression of eight selected TF genes in developing heads of SB, LB, PI, PL and AC at six stages. *Error bars* represent standard deviation calculated from two biological replicates where each biological replicate contains a homogenate of six heads. **b** Relative expression ratios were subjected to an analysis of variance (ANOVA) to test for expression differences among five Arctic charr morphs and six stages (*M* morph; *T* stage (time); *MxT* morphs by time effects; *P* values of <0.05, 0.01 and 0.001 are indicated by *one*, *two* and *three asterisks*, respectively). Subsequently, a post hoc Tukey’s HSD test was performed to analyse the expression of candidates among the morphs. *Green* to *red* colour gradient of morphs represents low to high expression levels and morphs with no *connecting line* have significantly different expression (*α* = 0.05). The *bold* and *underlined* gene(s) displayed distinct benthic–limnetic expression dynamics

With the objective of finding potential upstream regulatory pathway(s) and/or TFs influencing the co-expressed genes, we selected the three genes, *Chd4, Cspp1* and *Dlg1,* with persistent and strong reduction in expression in developing heads of benthic morphs and with positive co-expression in mammals and Arctic charr. We retrieved a list of over 200 genes showing co-expression in mammals with these three genes (Additional file 3: Table S3). This list was used as input for a knowledge-based TF enrichment analysis in human and mouse using WebGestalt v2 [34]. In both human and mouse, binding sites belonging to 17 TFs were significantly overrepresented on promoters of the input genes (Additional file 4: Table S4). We chose the eight most significantly enriched TFs for further gene expression analysis (Fig. 4a). The results revealed differential expression of all the eight TFs over time and seven TFs between the morphs (Fig. 4b). However, only two TFs (*Ahr2b* and *Ap2*) were differentially expressed between benthic and limnetic charr, with higher levels of expression for *Ahr2b* in benthic morphs and higher expression of *Ap2* in limnetic morphs. This implicates *Ahr2b* and *Ap2* as potential transcriptional regulators (i.e. transcriptional repressor and activator, respectively) of the co-expressed genes identified above.

### Differentially expressed AhR target genes show transcriptional correlation with the previously identified genes

**Fig. 5:**
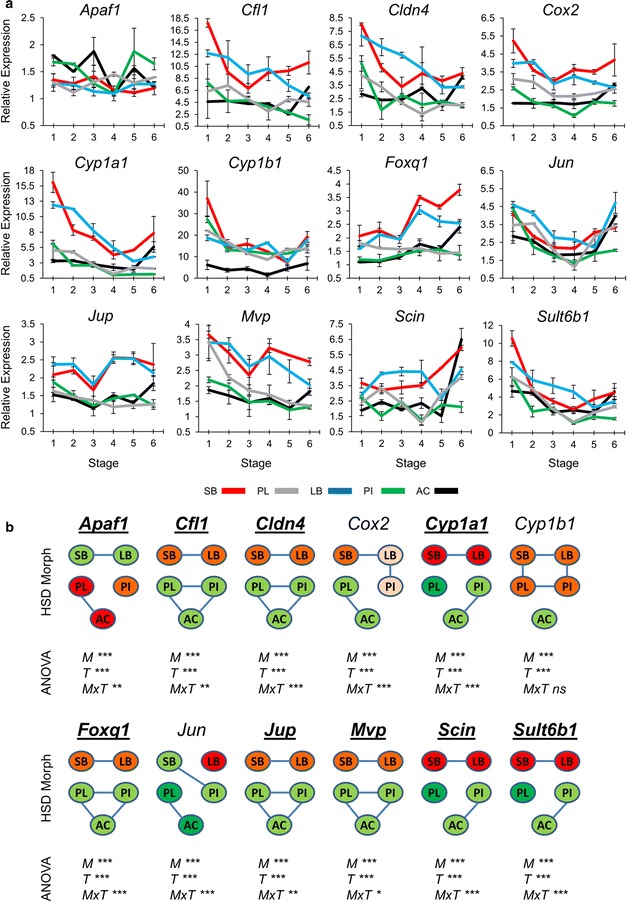
Differential expression of AhR-regulated genes in developing heads of Arctic charr morphs. **a** Relative expression of twelve AhR-regulated candidate genes in developing heads of SB, LB, PI, PL and AC at six stages. *Error bars* represent standard deviation calculated from two biological replicates where each biological replicate contains a homogenate of six heads. **b** Relative expression ratios were subjected to an analysis of variance (ANOVA) to test the expression differences amongst five Arctic charr morphs and six stages (*M* morph; *T* stage (time); *MxT* morphs by time effects; *P* values of <0.05, 0.01 and 0.001 are indicated by *one*, *two* and *three asterisks*, respectively). Subsequently, a post hoc Tukey’s HSD test was performed to analyse the expression of candidates among the morphs. *Green* to *red* colour gradient of morphs represents low to high expression levels and morphs with no *connecting line* have significantly different expression (*α* = 0.05). The *bold* and *underlined* gene(s) displayed differential benthic–limnetic expression dynamics

**Fig. 6:**
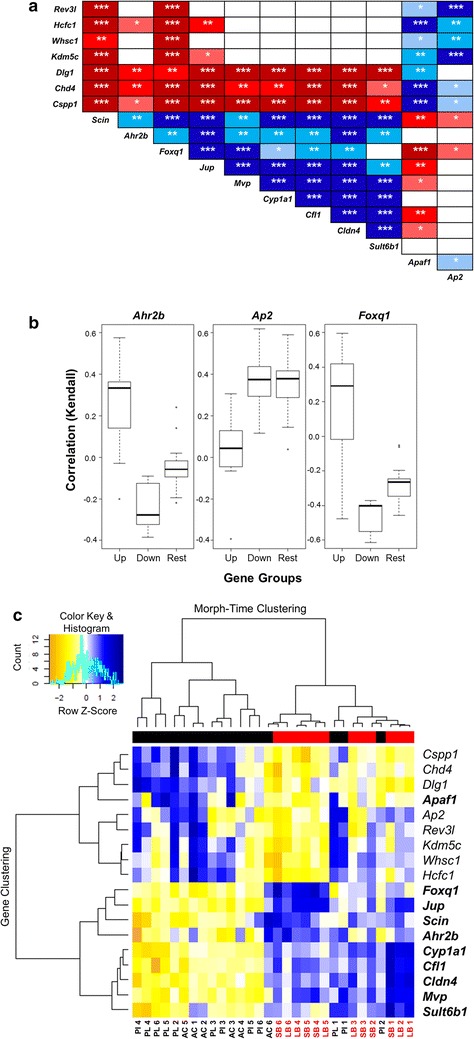
Significant expression correlations between *Ahr2b*, AhR-regulated genes and the seven previously identified genes and their distinct benthic–limnetic expression dynamics. **a** Correlation analyses revealed negative or positive expression correlation of *Ahr2*, AhR-regulated genes and the seven previously identified genes during craniofacial development. Pearson correlation coefficient (*r*) was used to assess the pairwise expression similarity between the candidate genes. *Blue shading* represents positive and *red shading* represents negative expression correlation and the colour gradients indicate correlation coefficients (*r*) above critical values (2-tail; *df* = 28). *P* values of <0.05, 0.01 and 0.001 are indicated by *one*, *two* and *three asterisks*, respectively. **b** Analyses of correlations (Kendall’s tau) between the expression of *Ahr2*, *Ap2* and *Foxq1* and the expression of other genes classified in three groups: an Up group including genes with significantly higher expression in the benthic charr morphs, a Down group with genes with significantly lower expression in benthic morphs and a Rest group including genes without significant expression difference between benthic and limnetic charr. Non-parametric Kruskal–Wallis ANOVA indicated difference among gene groups for the three TFs (*P* < 0.005). **c** A distinct morph and time-dependent expression pattern of the AhR-regulated and the seven previously identified genes were seen in developing heads of Arctic charr. The selected genes were subjected to double clustering based on expression correlation of the genes and between morphs by time effects across six stages. Both gene and morph-time clustering dendrograms include two main branches and each branch contains a few sub-clusters. A clear benthic–limnetic separation is observed in sub-clusters of the morph-time dendrogram (benthic samples depicted in *red*). Also, a clear separation of AhR-induced and the seven identified genes is observed in the gene clustering. *Blue* and *yellow shadings* represent higher and lower relative expression, respectively

The observations above led us to hypothesize that the signalling pathway mediated by aryl hydrocarbon receptors (AhR pathway) is involved in the emergence of distinct benthic–limnetic craniofacial morphologies. This hypothesis is also bolstered by the fact that a homozygous loss-of-function but viable mutant of an aryl hydrocarbon receptor (*Ahr2*) in zebrafish has a pointed snout and protruding lower jaw [65]. The phenotypic differences between the mutant and a wild-type zebrafish to some extent reflect the phenotypic dichotomy seen in Arctic charr, i.e. the long lower jaw and pointed snout of limnetic morphs versus the shorter lower jaw and blunt snout of benthic morphs [65]. In view of this, we selected twelve genes for expression analysis that are known downstream targets of the AhR pathway and also possibly involved in vertebrate craniofacial morphogenesis and/or skeletogenesis (Additional file 1: Table S1). Interestingly, the qPCR results indicated that eight of these genes had increased expression (and one gene had decreased expression) in the heads of benthic morphs (relative to limnetic morphs) during development (Fig. 5). The magnitude of the expression differences varied considerably among the genes with *Cyp1a1* and *Foxq1* showing the largest expression differences between morphs in several developmental time-points (Fig. 5a). We calculated Pearson’s correlation coefficients of the expression levels for AhR target genes showing distinct benthic–limnetic transcriptional dynamics together with the previously identified genes, over all morphs and time-points (Fig. 6a) and found strong positive expression correlation between pairs of AhR targets, and also between them and *Ahr2b*. On the other hand, the same genes showed negative expression correlation with genes identified in previous steps (mostly with *Chd4, Cspp1* and *Dlg1*) (Fig. 6a). Strikingly, the only AhR target gene with reduced expression in benthic morphs, *Apaf1,* displayed positive correlation in expression with all the genes found in former steps as well as with the *Ap2* gene (Fig. 6a). *Ap2* showed positive expression correlations with the set of genes that had higher expression in limnetic heads, but negative expression correlations with *Scin* and *Foxq1,* which is itself an AhR-regulated transcription factor.

To further assess expression associations between the TFs and the other genes studied here, we calculated the correlation between expression levels of all the genes together and the TFs (Fig. 6b, Additional file 5: Figure S1). The genes were classified into three categories, “Up”, “Down” and “Rest” based on significant expression differences between benthic and limnetic morphotypes. Genes in the Up group had higher expression in benthic charr, while the Down group showed lower expression in benthic charr. The Rest category consists of genes with no significant expression differences between the two morphotypes. As was predicted, *Ahr2b* showed significant positive correlation with its target genes (Up-genes in Fig. 6b) and a negative, but weak relationship with the Down-genes group. *Ahr2b* levels did not associate on average with genes in the Rest group. The Down genes were positively correlated with six of the other candidate regulators (*Ap2*, *E2f1*, *Maz*, *Nrf1*, *Sp1* and *Yy1*) (Fig. 6b, Additional file 5: Figure S1), of which only *Ap2* differed significantly between the morphotypes. The opposite effect, i.e. negative correlation with the Up genes, was generally not observed for these other TFs. Curiously, however, these factors all showed positive correlation with genes of the Rest category. The correlation of *Foxq1* expression with the three groups of genes was similar to that seen for *Ahr2b*, except *Foxq1*, which is a TF known to be activated by AhR signalling, showed stronger negative association with the Down genes (Fig. 6b).

To summarize the overall patterns of expression, we conducted double hierarchical clustering of relative expression values of genes vs. morph and time (Fig. 6c). The genes are divided into two main branches. The AhR targets and *Ahr2b* (in bold) clustered in one branch and the rest of the genes with reduced expression in benthic morphs (including *Ap2* and *Apaf1*) clustered in the other branch. Predictably, the clustering of samples (morph-time) revealed a separation of morphotypes with benthic morphs (red bar) on one of the two main branches and the limnetic morphs (black bar) on the other. The exceptions to this were PL and PI at early time-points, and AC which clustered with the benthic samples at the last time-point (Fig. 6c). Taken together, these results suggest higher activity in the AhR signalling pathway, probably through *Ahr2b*, during the craniofacial development of benthic Arctic charr morphs.

### Craniofacial expression patterns of differentially expressed genes

**Fig. 7:**
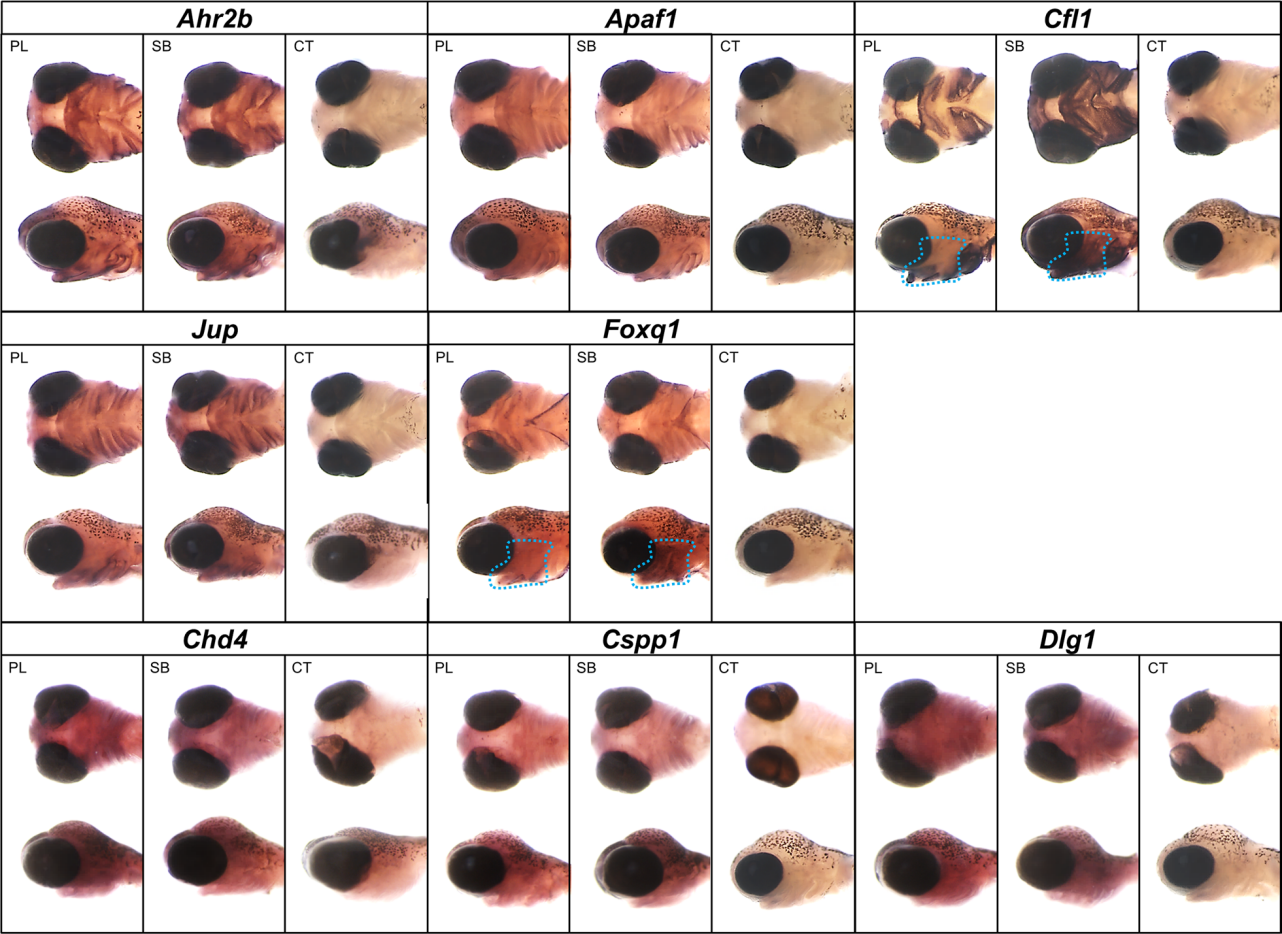
Craniofacial expression patterns of candidate genes in developing heads of two contrasting Arctic charr morphs (SB and PL). In situ hybridization shows the overlapping expression pattern of *Ahr2b*, *Apaf1*, *Cfl1*, *Foxq1* and *Jup* at the 238 (*τ*_s_) time-point. *Ventral* and *lateral views* indicate the overlapping expression of the genes in the area surrounding the mouth, lower jaw and pharyngeal arches. An extended expression pattern is observed for *Cfl1* and *Foxq1* in ventral facial elements and pharyngeal arches of SB morph (*marked area*). Furthermore, *Cfl1* had higher expression in the fronto-nasal region in SB (*marked area*). *Chd4*, *Cspp1* and *Dlg1* showed diffuse expression in most parts of the head mesenchyme without noticeable spatial expression differences between the two morphs

To determine the spatial craniofacial expression and differences in expression pattern between benthic and limnetic morphs, we investigated in situ expression of eight genes. In addition to the differentially expressed *Ahr2b*, we selected the three AhR-regulated genes with higher expression level in the benthic morphs (*Cfl1, Foxq1* and *Jup*) and four genes with reduced expression in the benthic morphs (*Apaf1*, *Chd4*, *Cspp1* and *Dlg1*). The spatial expression pattern of these genes was studied in two contrasting morphs (PL and SB) at time point 238 *τ*_s_ during early craniofacial skeletal morphogenesis. In concordance with the correlation analyses, all seven genes showed craniofacial expression (Fig. 7). A similar expression pattern could be observed for the selected genes in developing heads of both PL and SB. Five of the genes, *Ahr2b*, *Apaf1*, *Cfl1*, *Foxq1* and *Jup*, had pronounced expression in regions surrounding the mouth, ventral and ventrolateral facial elements and pharyngeal arches. In the case of *Cfl1* and *Foxq1*, which showed large expression differences between the morphs in the qPCR analyses (Fig. 5), a more pronounced and extended expression was detectable in ventral facial elements and pharyngeal arches of the SB morph (marked area in Fig. 7). The most evident spatial differences in expression were observed for *Cfl1*, where in the SB morph the expression was extended all over the region encompassing frontonasal region, the lower jaw, ventral facial elements and pharyngeal arches (Fig. 7). The expression of *Chd4*, *Cspp1* and *Dlg1* did not show clear spatial patterns, but was diffused all over the head mesenchyme without any noticeable spatial differences between the morphs.

## Discussion

An increasing number of gene expression studies have been launched to unravel the molecular mechanisms underlying adaptive morphological novelties in feeding structures of a variety of fish species [19–23, 27, 28, 66, 67]. Developmental shifts in the relative timing of skeletal gene expression, i.e. *collagen* genes, have been shown to underpin the radiation of feeding structures in Antarctic notothenioid fish along the benthic–limnetic axis of habitat [22]. Similarly, a heterochronic shift in developmental expression of a *Calmodulin* gene has been suggested to drive the variable growth rates of the jaws across Belonoid fishes [23]. In addition to the heterochrony in expression, the developmental differences in the level of gene expression have been repeatedly linked to occurrence of adaptive skeletal changes in teleost fish [19–21, 28, 68, 69]. It is important to note that a highly similar skeletal phenotype might emerge from the altered expression of different regulatory genes as two distinct transcriptional mechanisms give rise to the loss of pelvic skeleton in stickleback and fugu [69, 70]. Studies in cichlids have shown that genetic changes in regulatory elements may account for altered expression of genes involved in skeletogenesis and the subsequent morphological variations [20, 71, 72]. Also, external stimulants during early development, such as mechanical strain in bone, can alter the expression of skeletal genes (e.g. ECM genes) and result in adaptive phenotypic plasticity in feeding structures [27, 66]. The molecular basis of adaptive morphological novelties in feeding structures can be further elucidated through the expression analysis of functionally and transcriptionally related gene networks. The identification of such gene network provides a promising foundation to investigate potential upstream regulatory elements and interacting signalling pathways [26, 29, 30]. The related examples include the identification of gene expression networks associated with evolution of oral jaw dentition [73] and pharyngeal jaw developmental plasticity [27] in East African cichlids, as well as craniofacial skeletal diversity in sympatric Arctic charr [19] and lake whitefish [28].

Recently, we identified a gene network involved in ECM organization, cell-substrate adhesion and skeletogenesis with higher levels of expression in developing heads of benthivorous Arctic charr than in limnetic morphs [19]. In the same study, we predicted several transcription factors, such as *Ap1* and *Ets2*, as potential regulators of the network. Although these TFs could be key elements causing the induction of the ECM-related gene network, they are likely to be regulated through pathways further upstream. Such upstream pathways might also regulate other gene networks involved in craniofacial morphogenesis. In general, the activation of a particular signalling pathway leads to both induction and repression of different sets of downstream genes during embryonic development [29]. Therefore, we set out to search for co-expressed genes involved in skeletogenesis/craniofacial formation focusing on genes that had higher expression during head development in limnetic than in benthic morphs of Arctic charr. Such genes may be a part of networks that are transcriptionally linked to the previously described ECM-related gene network through common upstream regulatory elements.

In an iterative selection for co-expressed genes, we found seven candidate genes, *Chd4*, *Cspp1, Dlg1, Hcfc1, Kdm5c,Rev3* *l* and *Whsc1*, showing reduced expression in benthic morphs. In a recent study on mammalian embryonic cells, *Chd4* has been shown to be a potent co-repressor of sry-box containing gene 9 (*Sox9*), a highly conserved gene involved in skeletal development [47]. While the role of *Chd4* in craniofacial development has not been investigated, other genes such as *Cspp1,Dlg1,Hcfc1* and *Whsc1* are well known to be essential for normal development of craniofacial skeletal elements [55–57, 59]. In our study, the positive expression correlation of these genes could suggest their co-regulation through shared transcriptional regulatory elements during Arctic charr head development. Using co-expression data from mammals [33], we found over 200 genes to be co-expressed with *Chd4*, *Cspp1* and *Dlg1* as well as with at least one of the other identified genes. Binding sites for 17 TFs were significantly enriched on the promoter regions of these genes in human and mouse. By profiling the expression of eight predicted TFs with most significantly overrepresented binding sites, we found two of them, *Ahr2b* and *Ap2*, to be differentially expressed between benthic and limnetic morphotypes. Curiously, a zebrafish *Ahr2* mutant displays an extension of the ethmoid and mandibular regions, shallower head and longer snout which is a reminiscent phenotype of limnetic Arctic charr morphology [16, 65]. Furthermore, *Ap2*-dependent signals from epithelium have been demonstrated to induce skeletogenesis during craniofacial development in zebrafish [74]. It is unclear, however, whether *Ahr2* and *Ap2* show a direct transcriptional interaction during development.

Aryl hydrocarbon receptor (*Ahr*) belongs to the basic-helix-loop-helix (bHLH)/Per-Arnt-Sim (PAS) family of heterodimeric transcriptional regulators and it was originally described as a receptor that mediates many of the toxic effects of environmental contaminants of the dioxin family [75]. It is highly unlikely that the actual role of AhR is to sense environmental contaminants, but the search for endogenous ligands is still ongoing (reviewed in [76]). There is, however, increasing evidence that the AhR pathway plays important roles in a variety of developmental, physiological and immunological processes [75, 77–81]. The pathological effects of AhR pathway induction in zebrafish are exerted mostly through the transcriptional regulation of a large number of downstream genes [82]. It is also known that the activation of the AhR pathway not only alters the expression of ECM remodelling genes, but the pathway itself can also be affected by changes in ECM organization and cell-substrate adhesion [83]. Many of the identified TF binding sites in this study (such as binding sites for *Ahr, Ap2, E2f1, Max, Myc, Myb, Nf1, Sp1, Usf, and Yy1*) (Additional file 4: Table S4) are also predicted on the promoter of AhR-regulated genes in mammals [78, 84]. This suggests that these TFs cooperate in the regulation of AhR target genes that could be conserved across vertebrates. To further investigate the involvement of the AhR pathway, we examined the transcriptional dynamics of 12 downstream targets of the pathway which were selected based on existing literature of their craniofacial expression pattern and/or their role in vertebrate skeletal formation (Additional file 1: Table S1). All of these candidates, except *Apaf1*, are known to be induced by activation of the AhR pathway. Accordingly, we found higher expression of most candidates in developing heads of benthic morphs, whereas only *Apaf1* displayed reduced expression relative to limnetic morphs (known to be down regulated by AhR). Most of the AhR-regulated candidates as well as *Ahr2b* had negative expression correlation with the seven genes identified at the beginning of this study and only *Apaf1* showed positive expression correlations with them (Fig. 6a). The AhR-regulated candidates also had positive expression correlation with the previously identified co-expression network of ECM genes during the same developmental time-points ([19], data not shown). These results suggest higher activity of the AhR pathway during head development of benthic than limnetic morphs.

In Atlantic salmon, multiple aryl hydrocarbon receptor genes are reported, four of which are paralogues of *Ahr2*, with markedly higher expression than the two paralogues of *Ahr1* [85]. Here, we only report results for one *Ahr2* paralogue (*Ahr2b*) which had the highest expression level in a developmental transcriptome of Arctic charr [36]. Two other paralogues (*Ahr2a* and *Ahr2c*) were not detected in the transcriptome data, whereas the third one (*Ahr2d*) was identified with lower expression but qPCR analysis with paralogue specific primers showed no differences in expression between benthic and limnetic morphs (data not shown). Developmental effects of AhR pathway activation on skeletogenesis are mediated by *Ahr2* in zebrafish and transcriptional suppression of *Ahr2* could partially rescue the resulting inhibition of jaw chondrogenesis during development [86]. Analysis of the non-functional *Ahr2* mutant in zebrafish illustrated the endogenous role of the AhR pathway in craniofacial development [65], but the molecular mechanism for this craniofacial effect has remained unclear. So far, investigation of potential downstream targets in zebrafish identified a contribution of *Sox9* to AhR-induced jaw malformation [87]. In a previous study, we did not detect such differential expression of *Sox9* at early stages of craniofacial skeletal formation in different Arctic charr morphs [40]. In zebrafish, down-regulation of the *Sox9* isoform (*Sox9b*) does not occur immediately after induction of AhR pathway which might suggest an indirect mechanism for AhR regulation of *Sox9b* [87]. It has been reported that *Ap2* negatively regulates chondrocyte differentiation by suppressing *Sox5/6*, but not *Sox9* [88]. In contrast to *Ahr2b*, the higher expression of *Ap2* in limnetic morphs and the reduced jaw skeleton and shorter snout of *Ap2* mutant in zebrafish [65, 74] suggest counteracting roles for *Ap2* and *Ahr2b* in mediating differential effects on craniofacial skeletogenesis.

*Cyp1a1* is known as the most potently induced AhR target [75], and its expression has been identified in craniofacial tissues of teleost fish species [89]. In our study, *Cyp1a1* shows strong benthic–limnetic expression differences (Fig. 5). A functional study revealed that suppression of *Cyp1a1* expression does not prevent craniofacial malformation induced by activation of AhR during zebrafish development [90]. This demonstrates that even though induction of *Cyp1a1* is an indicator of AhR pathway activation, it is not the key target of the pathway mediating the effects on craniofacial skeletal formation.

Among the other AhR downstream targets which could be involved in mediating the craniofacial effects (Additional file 1: Table S1), we found 8 more genes showing benthic–limnetic differential expression over the examined developmental period (Fig. 5). Three of the genes, *Apaf1*, *Cfl1* and *Foxq1*, might be of importance for the development of the craniofacial characteristics induced by AhR pathway activity. *Apaf1* is an evolutionarily conserved cytosolic protein with a critical role in regulation of developmental apoptosis in mammals [91] and we found that its expression is higher in developing heads of limnetic morphs. A non-functional *Apaf1* mutant in mice displays defective craniofacial development through altered regulation of hedgehog (Hh) signalling [92]. The Hh pathway itself is known to be a key driver for adaptive variation of craniofacial skeletal structures in cichlids [93]. In mammalian cells, the expression of *Apaf1* was shown to be inhibited by overexpression of *Ahr* through a complex formed by AhR and E2f1 which binds to the *Apaf1* promoter at a region containing E2f1 binding site, but no AhR binding sites [94]. In general, AhR can act as a co-repressor of E2f-dependent transcription, and this is particularly interesting because binding sites for E2f were significantly enriched in our TF overrepresentation analysis [95].

*Cfl1* encodes a protein (cofilin-1, non-muscle) that plays a role in the regulation of cell morphology and cytoskeletal organization by affecting the polymerization of actin [96]. Activation of the AhR pathway can induce the expression of *Cfl1* [97]. Moreover, loss of function mutations as well as knock-down of *Cfl1* in zebrafish result in morphological changes in the lower jaw and pharyngeal arches [96]. Our study showed that expression of *Cfl1* was consistently higher in developing heads of the benthic morphs and its expression correlated negatively with *Apaf1*. Moreover, WISH analysis showed clear differences in the spatial expression pattern between the morphotypes, i.e. in the frontonasal region and in components of the lower jaw and pharyngeal arches (Fig. 7).

In zebrafish, an isoform of the transcription factor *Foxq1* (*Foxq1b*) has been reported to be induced by activation of *Ahr2* and its expression was limited to the jaw primordium [98]. Our results show higher expression of *Foxq1* in developing heads of benthic morphs and strong negative expression correlation with *Apaf1* (Figs. 5, 6a). We also identified differences in levels and spatial patterns of expression for *Foxq1* between contrasting morphs in regions limited to ventral craniofacial structures, where it overlaps with the expression of *Ahr2b*, *Apaf1*, and *Cfl1* (Fig. 7). Taken together, the transcriptional dynamics of *Apaf1, Cfl1* and *Foxq1* indicate higher AhR activity in benthic morphs and suggest a role for these three genes in divergent craniofacial morphogenesis in Arctic charr.

The other 5 AhR-regulated genes tested in this study, *Cldn4, Jup, Mvp, Scin* and *Sult6b1*, were found to be more highly expressed in benthic morphotypes (Fig. 5). Furthermore, most of these genes showed positive expression correlation with *Ahr2b* suggesting *Ahr2b*-dependent regulation. A junction plakoglobin gene, *Jup*, encodes a protein involved in cadherin-mediated intercellular communication [99]. In mammalian cells, *Jup* shows an interesting biphasic transcriptional response to AhR activity [100]. While high doses of AhR pathway agonists repressed *Jup* expression, a low dose could induce *Jup* expression [100]. Therefore, the increased expression of *Jup* in developing heads of benthic morphotypes might indicate moderate AhR pathway activity. Another interesting gene, *Scin* (Adseverin), was already shown in a previous transcriptome analysis to be among the top differentially expressed genes between two contrasting morphs [36]. *Scin* plays a role in rearranging the actin cytoskeleton, chondrocyte differentiation and in skeletal formation [101, 102]. In mice, *Scin* has been shown to be a direct target of the AhR pathway [103]. Additional studies are required to determine whether the genes found in this study have role in phenotypic variation of vertebrate craniofacial morphogenesis and whether and to what extent their transcription is regulated by the AhR pathway during skeletal development.

The differential transcriptional signatures of the selected AhR target genes clearly correlate with the differences in craniofacial development between charr morphotypes. However, further studies are required to differentiate between expression dynamics of the paralogues of each AhR target gene to identify the potential paralogue-specific response to the pathway. The quantified expression differences are most likely caused by genetic differences that affect regulators (TF’s, miRNAs, translation regulators or *cis*-elements) of the AhR pathway or parallel pathways. Can such differences in the developmental circuitry of craniofacial development play a role in the evolution of the charr morphs? Phenotypes and alleles of developmental genes can be influenced by genetic background [104, 105]. An interesting and highly relevant example is the fact that different inbred strains of mice display differential response to AhR-mediated effects on mandible development [106]. The morphs studied here all (except AC) exist in sympatry in Lake Thingvallavatn. Coalescent simulations involving PL and SB charr from the lake suggest a scenario of rapid divergence in allopatry followed by very slow (large populations and slow drift) divergence in sympatry and very low gene flow among the morphs [15]. Although the nature of the genetic separation (and possibly reproductive barriers) is unknown, our data on craniofacial development in hybrids of PL and SB charr suggested developmental incompatibilities resulting in smaller heads and narrowing of the mouth and pharyngeal tract in the hybrid offspring (Kapralova et al., unpublished data). This could reduce the fitness of hybrids (e.g. at the start of exogenous feeding), and lead to strong selection against hybrids. In this respect, it is tempting to speculate that the initial, rapid divergence processes postulated could involve changes in key developmental pathways of craniofacial development, possibly the AhR pathway, resulting in developmental incompatibility of hybrids, a situation that would select for further reproductive isolation during the sympatric phase.

## Conclusions

In this study, we investigate the molecular mechanism underlying craniofacial divergence in the highly polymorphic Arctic charr of Lake Thingvallavatn (Iceland). We have found a network of co-expressed genes with higher expression in limnetic than benthic morphotypes during early stages of craniofacial development. Key members of the network and their genetic interactions are conserved across vertebrate species. Searching for predicted upstream TFs regulating the network, we identified a receptor of the AhR pathway, *Ahr2b* and found a set of AhR-regulated genes to be differentially expressed between the morphotypes. We also confirmed an overlapping expression pattern of *Ahr2b* and AhR target genes in craniofacial structures of Arctic charr embryos. Taken together, this study suggests the AhR pathway as a key modulator of transcriptional differences along the benthic–limnetic axis of craniofacial development in Arctic charr. Considering the conserved nature of the network, it is likely to have a much broader relevance in the early development of craniofacial structures in vertebrates.

## Additional files

**Additional file 1: Table S1.** AhR-regulated candidate genes and available literature on their response to AhR activation, expression during craniofacial development and involvement in skeletal formation.
**Additional file 2: Table S2.** Primers designed for cDNA cloning and qPCR study of Arctic charr.
**Additional file 3: Table S3.** *Chd4*-*Cspp1*-*Dlg1* co-expressed genes in humans and mice retrieved from COXPRESdb. More than 200 genes were identified to have co-expression with *Chd4*, *Cspp1* and *Dlg1* after setting the mutual rank (MR) to the top-ranked 2000 genes and the reliability score of 1 to 3.
**Additional file 4: Table S4.** Transcription factor enrichment analysis using WEB-based GEne SeT AnaLysis Toolkit (WebGestalt v2). The list of more than 200 genes co-expressed with *Chd4*, *Cspp1* and *Dlg1* in human and mice are used as input. (Cut-off criteria: p < 0.001 adjusted with hypergeometric statistical analysis; Bonferroni multiple test adjustment).
**Additional file 5: Figure S1.** Analyses of correlations between the expression of TFs (*E2f1*, *Yy1*, *Nrf1*, *Sp1*, *Maz* and *Myc*) and expression of the candidate target genes in the study classified in three groups. The Up group indicates genes with significantly higher expression in the benthic charr morphs, Down includes genes with significantly lower expression in benthic morphs and Rest constitutes genes without significant expression difference between benthic and limnetic charr. The boxplots summarize Kendall correlation coefficients by gene groups. Non parametric Kruskal–Wallis ANOVA indicated differences among gene groups for all TFs except *Myc* (*P* = 0.015 for *Sp1*, *P* < 0.005 for the other four genes).

## Abbreviations

AC: aquaculture limnetic-like fish stock
Cq: quantification cycle
ECM: extracellular matrix
AhR: aryl hydrocarbon receptor pathway
LB: large benthivorous charr
PI: piscivorous charr
PL: planktivorous charr
RQ: relative expression quantity
SB: small benthivorous charr
TF: transcription factor
*τ*_s_: tau-somite relative age
