# Supplementary material for: Differential expression of the aryl hydrocarbon receptor pathway associates with craniofacial polymorphism in sympatric Arctic charr
Source: EvoDevo. 2015 Sep 16;6:27. doi: 10.1186/s13227-015-0022-6 (PMC4574265; doi:10.1186/s13227-015-0022-6)
Supplement: Supplementary file 1 — Additional file 1: Table S1. AhR-regulated candidate genes and available literature on their response to AhR activation, expression during craniofacial development and involvement in skeletal formation. [file 13227_2015_22_MOESM1_ESM.pdf]

Additional file 1

Gene Symbol	Description	Craniofacial Expression in Fish	Vertebrate Skeletal Formation	Response to AHR activation	References
Apaf1	Apoptotic Peptidase Activating Factor 1	?	Yes	Repression	[1–3]
Cfl1	Cofilin 1 (Non-Muscle)	Yes	Yes	Induction	[4–6]
Cldn4	Claudin 4	Yes	Yes	Induction	[7–9]
Cox2	Cyclooxygenase 2/ Prostaglandin-Endoperoxide Synthase 2 (PTGS2)	Yes	Yes	Induction	[10–12]
Cyp1a1	Cytochrome P450, Family 1, Subfamily A, Polypeptide 1	Yes	Yes	Induction	[7, 13–15]
Cyp1b1	Cytochrome P450, Family 1, Subfamily B, Polypeptide 1	Yes	?	Induction	[7, 16]
Foxq1	Forkhead Box Q1/ Hepatocyte Nuclear Factor 3 Forkhead Homolog 1 (HFH1)	Yes	?	Induction	[7]
Jun	Jun Proto-Oncogene/ Activator Protein 1 (AP1)	Yes	Yes	Induction	[17–19]
Jup	Junction Plakoglobin/ Desmoplakin-3	Yes	?	Induction/ Repression	[17, 20]
Mvp	Major Vault Protein	Yes	?	Induction	[21, 22]
Scin	Scinderin/ Adseverin	Yes	Yes	Induction	[23–26]
Sult6b1	Sulfotransferase Family, Cytosolic, 6B, Member 1	Yes	?	Induction	[7, 22]

References

1. Marlowe JL, Fan Y, Chang X, Peng L, Knudsen ES, Xia Y, Puga A: **The Aryl Hydrocarbon Receptor Binds to E2F1 and Inhibits E2F1-induced Apoptosis**. *Mol Biol Cell* 2008, **19**(August):3263–3271.

2. Cecconi F, Alvarez-bolado G, Meyer BI, Roth KA, Gruss P, Gene A: **Apaf1 (CED-4 Homolog) Regulates Programmed Cell Death in Mammalian Development Generation of a Null Allele of the Murine**. *Cell* 1998, **94**:727–737.

3. Long AB, Kaiser WJ, Mocarski ES, Caspary T: **Apaf1 apoptotic function critically limits Sonic hedgehog signaling during craniofacial development.** *Cell Death Differ* 2013, **20**:1510–20.

4. Huang L, Wang C, Zhang Y, Li J, Zhong Y, Zhou Y, Chen Y, Zuo Z: **Benzo[a]pyrene exposure influences the cardiac development and the expression of cardiovascular relative genes in zebrafish (Danio rerio) embryos.** *Chemosphere* 2012, **87**:369–75.

5. Rauch, G.J., Lyons, D.A., Middendorf, I., Friedlander, B., Arana, N., Reyes, T., and Talbot WS: **Submission and Curation of Gene Expression Data**. *ZFIN Direct Data Submiss* 2003.

6. Hong D, Chen H-X, Yu H-Q, Liang Y, Wang C, Lian Q-Q, Deng H-T, Ge R-S: **Morphological and proteomic analysis of early stage of osteoblast differentiation in osteoblastic progenitor cells.** *Exp Cell Res* 2010, **316**:2291–300.

7. Planchart A, Mattingly CJ: **2,3,7,8-Tetrachlorodibenzo-p-dioxin Upregulates FoxQ1b in Zebrafish Jaw Primordium**. *Chem Res Toxicol* 2010, **23**:480–487.

8. Thisse, C., and Thisse B: **High Throughput Expression Analysis of ZF-Models Consortium Clones**. *ZFIN Direct Data Submiss* 2005.

9. Wongdee K, Pandaranandaka J, Teerapornpuntakit J, Tudpor K, Thongbunchoo J, Thongon N, Jantarajit W, Krishnamra N, Charoenphandhu N: **Osteoblasts express claudins and tight junction-associated proteins.** *Histochem Cell Biol* 2008, **130**:79–90.

10. Kraemer S a, Arthur K a, Denison MS, Smith WL, DeWitt DL: **Regulation of prostaglandin endoperoxide H synthase-2 expression by 2,3,7,8,-tetrachlorodibenzo-p-dioxin.** *Arch Biochem Biophys* 1996, **330**:319–28.

11. Grosser T, Yusuff S, Cheskis E, Pack M a, FitzGerald G a: **Developmental expression of functional cyclooxygenases in zebrafish.** *Proc Natl Acad Sci U S A* 2002, **99**:8418–23.

12. Li C-J, Chang J-K, Wang G-J, Ho M-L: **Constitutively expressed COX-2 in osteoblasts positively regulates Akt signal transduction via suppression of PTEN activity.** *Bone* 2011, **48**:286–97.

13. Iwata H, Stegeman JJ: **In situ RT-PCR detection of CYP1A mRNA in pharyngeal epithelium and chondroid cells from chemically untreated fish: involvement in vertebrate craniofacial skeletal development?** *Biochem Biophys Res Commun* 2000, **271**:130–7.

14. Incardona JP, Carls MG, Teraoka H, Sloan C a., Collier TK, Scholz NL: **Aryl Hydrocarbon Receptor–Independent Toxicity of Weathered Crude Oil during Fish Development**. *Environ Health Perspect* 2005, **113**:1755–1762.

15. Incardona JP, Day HL, Collier TK, Scholz NL: **Developmental toxicity of 4-ring polycyclic aromatic hydrocarbons in zebrafish is differentially dependent on AH receptor isoforms and hepatic cytochrome P4501A metabolism.** *Toxicol Appl Pharmacol* 2006, **217**:308–21.

16. Yin H-C, Tseng H-P, Chung H-Y, Ko C-Y, Tzou W-S, Buhler DR, Hu C-H: **Influence of TCDD on zebrafish CYP1B1 transcription during development.** *Toxicol Sci* 2008, **103**:158–68.

17. Thisse, B., Thisse C: **Fast Release Clones: A High Throughput Expression Analysis**. *ZFIN Direct Data Submiss* 2004.

18. Puga A, Nebert DW, Carrier F: **Dioxin induces expression of c-fos and c-jun proto-oncogenes and a large increase in transcription factor AP-1.** *DNA Cell Biol* 1992, **11**:269–81.

19. McCabe LR, Kockx M, Lian J, Stein J, Stein G: **Selective Expression of fos- and jun-Related Genes during Osteoblast Proliferation and Differentiation**. *Exp Cell Res* 1995, **218**:255–262.

20. Procházková J, Kozubík A, Machala M, Vondráček J: **Differential effects of indirubin and 2,3,7,8-tetrachlorodibenzo-p-dioxin on the aryl hydrocarbon receptor (AhR) signalling in liver progenitor cells.** *Toxicology* 2011, **279**:146–54.

21. Handley-Goldstone HM, Grow MW, Stegeman JJ: **Cardiovascular gene expression profiles of dioxin exposure in zebrafish embryos.** *Toxicol Sci* 2005, **85**:683–93.

22. Thisse, B., Pflumio, S., Fürthauer, M., Loppin, B., Heyer, V., Degrave, A., Woehl, R., Lux, A., Steffan, T., Charbonnier, X.Q. and Thisse C: **Expression of the zebrafish genome during embryogenesis (NIH R01 RR15402)**. *ZFIN Direct Data Submiss* 2001.

23. Svensson C, Silverstone AE, Lai Z-W, Lundberg K: **Dioxin-induced adseverin expression in the mouse thymus is strictly regulated and dependent on the aryl hydrocarbon receptor.** *Biochem Biophys Res Commun* 2002, **291**:1194–200.

24. Vieira F a, Thorne M a S, Stueber K, Darias M, Reinhardt R, Clark MS, Gisbert E, Power DM: **Comparative analysis of a teleost skeleton transcriptome provides insight into its regulation.** *Gen Comp Endocrinol* 2013, **191**:45–58.

25. Jia S, Nakaya N, Piatigorsky J: **Differential expression patterns and developmental roles of duplicated scinderin-like genes in zebrafish.** *Dev Dyn* 2009, **238**:2633–40.

26. Nurminsky D, Magee C, Faverman L, Nurminskaya M: **Regulation of chondrocyte differentiation by actin-severing protein adseverin.** *Dev Biol* 2007, **302**:427–37.
