# Supplementary figures and images for: Differential expression of the aryl hydrocarbon receptor pathway associates with craniofacial polymorphism in sympatric Arctic charr
Source: EvoDevo. 2015 Sep 16;6:27. doi: 10.1186/s13227-015-0022-6 (PMC4574265; doi:10.1186/s13227-015-0022-6)

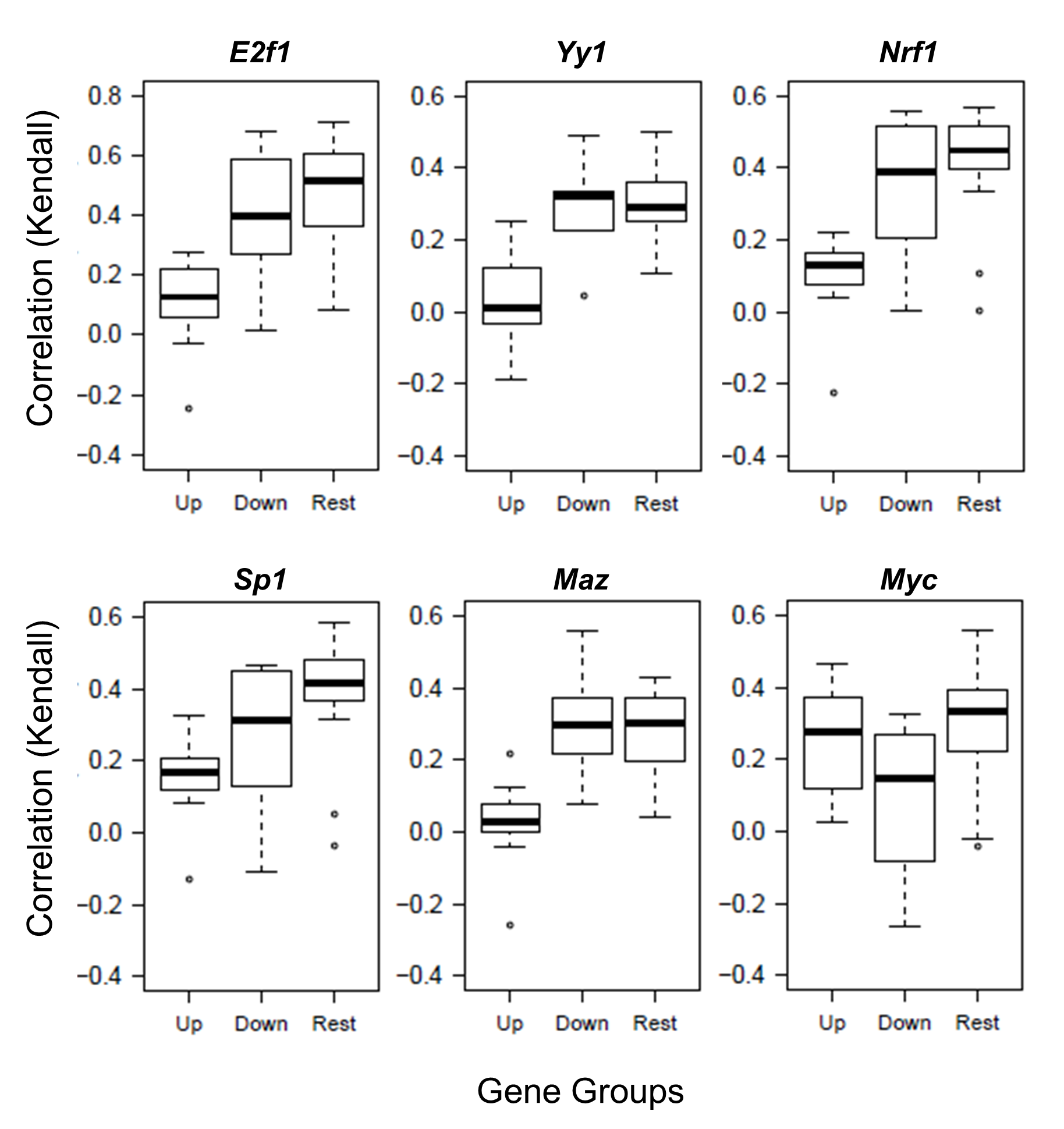

Supplement: Supplementary file 5 — Additional file 5: Figure S1. Analyses of correlations between the expression of TFs (E2f1, Yy1, Nrf1, Sp1, Maz and Myc) and expression of the candidate target genes in the study classified in three groups. The Up group indicates genes with significantly higher expression in the benthic charr morphs, Down includes genes with significantly lower expression in benthic morphs and Rest constitutes genes without significant expression difference between benthic and limnetic charr. The boxplots summarize Kendall correlation coefficients by gene groups. Non parametric Kruskal–Wallis ANOVA indicated differences among gene groups for all TFs except Myc (P = 0.015 for Sp1, P < 0.005 for the other four genes). [file 13227_2015_22_MOESM5_ESM.tif]
